# 4-{1-[4-(4-Bromo­phen­yl)-1,3-thia­zol-2-yl]-5-(4-fluoro­phen­yl)-4,5-dihydro-1*H*-pyrazol-3-yl}-5-methyl-1-(4-methyl­phen­yl)-1*H*-1,2,3-triazole

**DOI:** 10.1107/S1600536812024257

**Published:** 2012-05-31

**Authors:** Bakr F. Abdel-Wahab, Hanan A. Mohamed, Seik Weng Ng, Edward R. T. Tiekink

**Affiliations:** a Applied Organic Chemistry Department, National Research Centre, Dokki, 12622 Giza, Egypt; b Department of Chemistry, University of Malaya, 50603 Kuala Lumpur, Malaysia; c Chemistry Department, Faculty of Science, King Abdulaziz University, PO Box 80203 Jeddah, Saudi Arabia

## Abstract

In the title compound, C_28_H_22_BrFN_6_S, the central pyrazole ring has an envelope conformation, with the methine C atom being the flap atom. The dihedral angles between the least-squares plane through this ring and the adjacent thia­zole [18.81 (15)°] and triazole [1.83 (16)°] rings indicate a twist in the mol­ecule. A further twist is evident by the dihedral angle of 64.48 (16)° between the triazole ring and the attached benzene ring. In the crystal, C—H⋯N, C—H⋯F, C—H⋯π and π–π inter­actions [occurring between the thia­zole and triazole rings, centroid–centroid distance = 3.571 (2) Å] link mol­ecules into a three-dimensional architecture. The sample studied was a non-merohedral twin; the minor twin component refined to 47.16 (7)%.

## Related literature

For the biological activity of related compounds, see: Abdel-Wahab *et al.* (2009 ▶, 2012*a* ▶). For a related pyrazolyl-1,2,3-triazole structure, see: Abdel-Wahab *et al.* (2012*b* ▶). For the deconvolution of twinned data, see: Spek (2009 ▶).
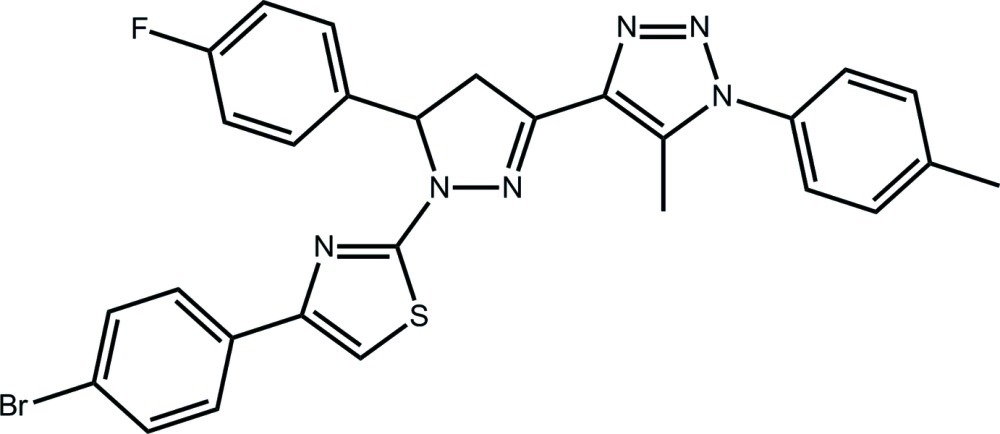


## Experimental

### 

#### Crystal data

C_28_H_22_BrFN_6_S
*M* *_r_* = 573.49
Orthorhombic, 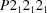

*a* = 11.3476 (7) Å
*b* = 14.0549 (8) Å
*c* = 15.954 (7) Å
*V* = 2544.5 (12) Å^3^
*Z* = 4
Mo *K*α radiation
μ = 1.74 mm^−1^
*T* = 100 K
0.40 × 0.20 × 0.10 mm

#### Data collection

Agilent SuperNova Dual diffractometer with an Atlas detector
Absorption correction: multi-scan (*CrysAlis PRO*; Agilent, 2011 ▶) *T*_min_ = 0.695, *T*_max_ = 1.000
7265 measured reflections
5293 independent reflections
4819 reflections with *I* > 2σ(*I*)
*R*_int_ = 0.029

#### Refinement

*R*[*F* ^2^ > 2σ(*F* ^2^)] = 0.035
*wR*(*F* ^2^) = 0.077
*S* = 1.02
5293 reflections
336 parameters
H-atom parameters constrained
Δρ_max_ = 0.36 e Å^−3^
Δρ_min_ = −0.50 e Å^−3^
Absolute structure: Flack (1983 ▶), 2028 Friedel pairs
Flack parameter: 0.001 (7)

### 

Data collection: *CrysAlis PRO* (Agilent, 2011 ▶); cell refinement: *CrysAlis PRO*; data reduction: *CrysAlis PRO*; program(s) used to solve structure: *SHELXS97* (Sheldrick, 2008 ▶); program(s) used to refine structure: *SHELXL97* (Sheldrick, 2008 ▶); molecular graphics: *ORTEP-3* (Farrugia, 1997 ▶) and *DIAMOND* (Brandenburg, 2006 ▶); software used to prepare material for publication: *publCIF* (Westrip, 2010 ▶).

## Supplementary Material

Crystal structure: contains datablock(s) global, I. DOI: 10.1107/S1600536812024257/su2440sup1.cif

Structure factors: contains datablock(s) I. DOI: 10.1107/S1600536812024257/su2440Isup2.hkl

Supplementary material file. DOI: 10.1107/S1600536812024257/su2440Isup3.cml

Additional supplementary materials:  crystallographic information; 3D view; checkCIF report

## Figures and Tables

**Table 1:** Hydrogen-bond geometry (Å, °) *Cg*1 is the centroid of the C23–C28 benzene ring.

*D*—H⋯*A*	*D*—H	H⋯*A*	*D*⋯*A*	*D*—H⋯*A*
C13—H13⋯N2^i^	1.00	2.58	3.488 (4)	151
C27—H27⋯F1^ii^	0.95	2.53	3.358 (4)	146
C8—H8*A*⋯*Cg*1^iii^	0.98	2.84	3.401 (3)	117

Symmetry codes: (i) 
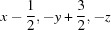
; (ii) 
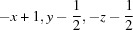
; (iii) 
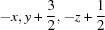
.

**Fig. 1.**
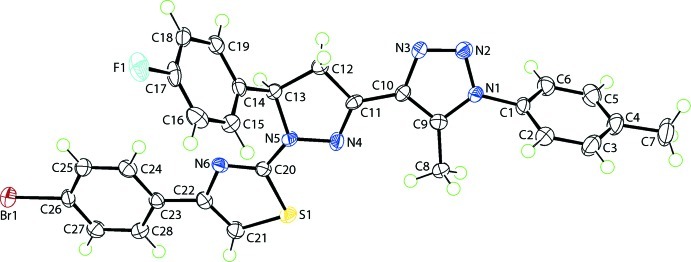
The molecular structure of (I) showing the atom-labelling scheme and displacement ellipsoids at the 70% probability level.

**Fig. 2.**
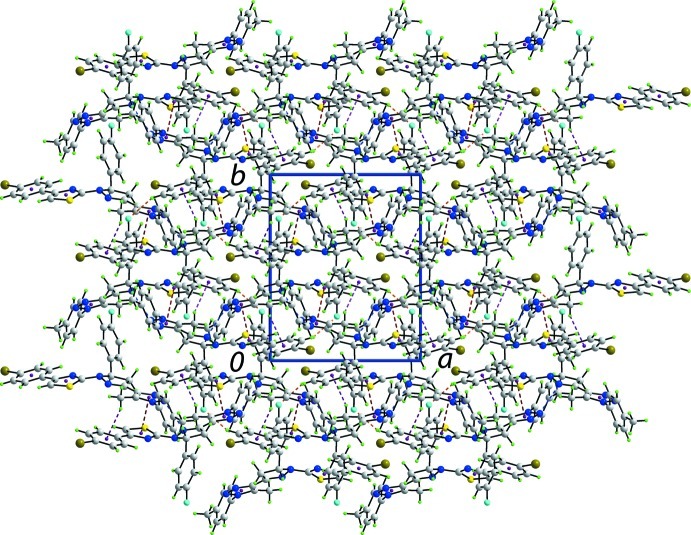
A view in projection down the c axis of the unit-cell contents for (I). The C—H···N, C—H···F, C—H···π and π—π interactions are shown as blue, orange, purple and brown dashed lines, respectively.

## supplementary crystallographic information

### Comment

The crystal structure determination of the title compound, 4-(4-bromophenyl)-2-(5-(4-fluorophenyl)-3-(5-methyl-1-*p*-tolyl-1*H*-1,2,3-triazol-4-yl)-4,5-dihydro-1*H*-pyrazol-1-yl)thiazole (I), was investigated in relation to the established biological activities exhibited by 3-(benzofuran-2-yl)-4,5-dihydro-5-phenyl-1-(4-phenylthiazol-2-yl)-1*H*-pyrazole and 1,2,3-triazol-4-yl-pyrazolylthiazoles (Abdel-Wahab *et al.* 2012*a*; Abdel-Wahab *et al.* 2009) and related structural studies (Abdel-Wahab *et al.*, 2012*b*).

The molecule of (I), Fig. 1, comprises a sequence of three linked five-membered rings with a benzene ring linked to each of these. The central pyrazole ring (r.m.s. deviation = 0.087 Å) adopts an envelope conformation with the methine-C13 atom being the flap atom. The molecule is twisted as seen in the dihedral angles between the least-squares plane through the pyrazole ring and the thiazole (r.m.s. deviation = 0.008 Å) and triazole (r.m.s. deviation = 0.004 Å) rings are 18.81 (15) and 1.83 (16)°, respectively. While the attached benzene ring to the thiazole ring is almost co-planar [dihedral angle = 7.00 (13)°], the benzene ring linked to the triazole ring is twisted out of its plane [dihedral angle = 64.48 (16)°].

In the crystal, C—H···N and C—H···π (Table 1), as well as π—π interactions occurring between the thiazole and triazole [inter-centroid distance = 3.571 (2) Å, angle of inclination = 5.08 (15)° for symmetry operation: -1/2 + *x*, 3/2 - *y*, -*z*] link molecules into layers in the *ac* plane. These layers are linked by C—H···F interactions (Fig. 2 and Table 1).

### Experimental

The title compound was prepared according to the reported method (Abdel-Wahab *et al.*, 2012*a*). Crystals were obtained from its DMF solution by slow evaporation at room temperature.

### Refinement

C-bound H-atoms were placed in calculated positions [N—H = 0.88 Å and C—H = 0.95 to 1.00 Å, *U*_iso_(H) = 1.2*U*_eq_(N,C) or = 1.5U_eq_(C-methyl)] and were included in the refinement in the riding model approximation. The sample studied is a non-merohedral twin and a full sphere of reflections was measured. As it was not possible to separate the diffraction spots in two domains, the twin domains were separated using the *TwinRotMat* routine of *PLATON* (Spek, 2009). The minor twin component refined to 47.16 (7)%. Two reflections, *i.e*. (-11 -8 2) and (0 -2 4), were omitted owing to poor agreement. The maximum and minimum residual electron density peaks of 1.44 and 0.53 e Å^-3^, respectively, were located 0.51 Å and 0.81 Å from the H13C and F9 atoms, respectively.

### Crystal data

**Table:**

C_28_H_22_BrFN_6_S	*F*(000) = 1168
*M**_r_* = 573.49	*D*_x_ = 1.497 Mg m^−^^3^
Orthorhombic, *P*2_1_2_1_2_1_	Mo *K*α radiation, λ = 0.71073 Å
Hall symbol: P 2ac 2ab	Cell parameters from 3622 reflections
*a* = 11.3476 (7) Å	θ = 2.6–27.5°
*b* = 14.0549 (8) Å	µ = 1.74 mm^−^^1^
*c* = 15.954 (7) Å	*T* = 100 K
*V* = 2544.5 (12) Å^3^	Prism, light-brown
*Z* = 4	0.40 × 0.20 × 0.10 mm

### Data collection

**Table:**

Agilent SuperNova Dual diffractometer with an Atlas detector	5293 independent reflections
Radiation source: SuperNova (Mo) X-ray Source	4819 reflections with *I* > 2σ(*I*)
Mirror monochromator	*R*_int_ = 0.029
Detector resolution: 10.4041 pixels mm^-1^	θ_max_ = 27.6°, θ_min_ = 2.6°
ω scan	*h* = −14→10
Absorption correction: multi-scan (*CrysAlis PRO*; Agilent, 2011)	*k* = −17→12
*T*_min_ = 0.695, *T*_max_ = 1.000	*l* = −20→11
7265 measured reflections	

### Refinement

**Table:**

Refinement on *F*^2^	Secondary atom site location: difference Fourier map
Least-squares matrix: full	Hydrogen site location: inferred from neighbouring sites
*R*[*F*^2^ > 2σ(*F*^2^)] = 0.035	H-atom parameters constrained
*wR*(*F*^2^) = 0.077	*w* = 1/[σ^2^(*F*_o_^2^) + (0.0325*P*)^2^ + 0.2812*P*] where *P* = (*F*_o_^2^ + 2*F*_c_^2^)/3
*S* = 1.02	(Δ/σ)_max_ = 0.001
5293 reflections	Δρ_max_ = 0.36 e Å^−^^3^
336 parameters	Δρ_min_ = −0.50 e Å^−^^3^
0 restraints	Absolute structure: Flack (1983), 2028 Friedel pairs
Primary atom site location: structure-invariant direct methods	Flack parameter: 0.001 (7)

### Special details

**Table:**

Geometry. All e.s.d.'s (except the e.s.d. in the dihedral angle between two l.s. planes) are estimated using the full covariance matrix. The cell e.s.d.'s are taken into account individually in the estimation of e.s.d.'s in distances, angles and torsion angles; correlations between e.s.d.'s in cell parameters are only used when they are defined by crystal symmetry. An approximate (isotropic) treatment of cell e.s.d.'s is used for estimating e.s.d.'s involving l.s. planes.
Refinement. Refinement of *F*^2^ against ALL reflections. The weighted *R*-factor *wR* and goodness of fit *S* are based on *F*^2^, conventional *R*-factors *R* are based on *F*, with *F* set to zero for negative *F*^2^. The threshold expression of *F*^2^ > σ(*F*^2^) is used only for calculating *R*-factors(gt) *etc*. and is not relevant to the choice of reflections for refinement. *R*-factors based on *F*^2^ are statistically about twice as large as those based on *F*, and *R*- factors based on ALL data will be even larger.

### Fractional atomic coordinates and isotropic or equivalent isotropic displacement parameters (Å^2^)

**Table:**

	*x*	*y*	*z*	*U*_iso_*/*U*_eq_	
Br1	0.23440 (3)	0.94410 (2)	−0.361341 (19)	0.02124 (8)	
S1	0.66530 (6)	0.86176 (5)	0.08004 (5)	0.01822 (17)	
F1	0.94395 (17)	1.26252 (12)	−0.25372 (13)	0.0294 (5)	
N1	1.2170 (2)	0.77775 (16)	0.21438 (15)	0.0152 (5)	
N2	1.2929 (2)	0.78394 (16)	0.14807 (16)	0.0174 (5)	
N3	1.2310 (2)	0.81441 (16)	0.08489 (15)	0.0154 (5)	
N4	0.9210 (2)	0.87452 (17)	0.06940 (16)	0.0171 (5)	
N5	0.8648 (2)	0.91513 (18)	0.00017 (16)	0.0170 (5)	
N6	0.6859 (2)	0.91897 (16)	−0.07364 (16)	0.0155 (5)	
C1	1.2602 (3)	0.7492 (2)	0.29537 (18)	0.0167 (6)	
C2	1.2594 (3)	0.8146 (2)	0.36070 (19)	0.0208 (6)	
H2	1.2265	0.8761	0.3532	0.025*	
C3	1.3073 (3)	0.7883 (2)	0.4366 (2)	0.0255 (7)	
H3	1.3062	0.8322	0.4818	0.031*	
C4	1.3573 (3)	0.6990 (2)	0.4485 (2)	0.0247 (7)	
C5	1.3549 (3)	0.6346 (2)	0.3825 (2)	0.0247 (8)	
H5	1.3869	0.5728	0.3902	0.030*	
C6	1.3067 (3)	0.6589 (2)	0.3059 (2)	0.0204 (7)	
H6	1.3055	0.6144	0.2611	0.024*	
C7	1.4158 (3)	0.6738 (3)	0.5301 (2)	0.0366 (9)	
H7A	1.3946	0.6086	0.5458	0.055*	
H7B	1.3895	0.7179	0.5739	0.055*	
H7C	1.5016	0.6785	0.5238	0.055*	
C8	1.0046 (3)	0.8013 (2)	0.2515 (2)	0.0178 (6)	
H8A	1.0048	0.7410	0.2823	0.027*	
H8B	0.9310	0.8072	0.2199	0.027*	
H8C	1.0112	0.8543	0.2912	0.027*	
C9	1.1057 (3)	0.8035 (2)	0.1929 (2)	0.0151 (6)	
C10	1.1170 (3)	0.8273 (2)	0.10935 (19)	0.0142 (6)	
C11	1.0306 (3)	0.8628 (2)	0.05071 (18)	0.0138 (6)	
C12	1.0619 (3)	0.8935 (2)	−0.03664 (19)	0.0170 (6)	
H12A	1.1013	0.8416	−0.0678	0.020*	
H12B	1.1138	0.9501	−0.0361	0.020*	
C13	0.9411 (2)	0.9169 (2)	−0.07472 (19)	0.0148 (6)	
H13	0.9171	0.8647	−0.1138	0.018*	
C14	0.9378 (2)	1.0115 (2)	−0.12096 (18)	0.0159 (6)	
C15	0.8894 (3)	1.0932 (2)	−0.0868 (2)	0.0196 (7)	
H15	0.8547	1.0910	−0.0326	0.024*	
C16	0.8913 (3)	1.1791 (2)	−0.1314 (2)	0.0241 (7)	
H16	0.8576	1.2353	−0.1085	0.029*	
C17	0.9427 (3)	1.1797 (2)	−0.2088 (2)	0.0208 (7)	
C18	0.9940 (3)	1.1004 (2)	−0.2437 (2)	0.0188 (7)	
H18	1.0310	1.1036	−0.2971	0.023*	
C19	0.9906 (3)	1.0161 (2)	−0.1994 (2)	0.0177 (6)	
H19	1.0247	0.9604	−0.2229	0.021*	
C20	0.7446 (3)	0.90135 (18)	−0.00607 (17)	0.0152 (6)	
C21	0.5397 (3)	0.8702 (2)	0.0193 (2)	0.0190 (7)	
H21	0.4623	0.8560	0.0382	0.023*	
C22	0.5671 (3)	0.8999 (2)	−0.0593 (2)	0.0171 (7)	
C23	0.4858 (2)	0.91170 (19)	−0.1308 (2)	0.0155 (6)	
C24	0.5272 (2)	0.9486 (2)	−0.20621 (19)	0.0180 (6)	
H24	0.6073	0.9676	−0.2105	0.022*	
C25	0.4538 (3)	0.9581 (2)	−0.27493 (19)	0.0197 (7)	
H25	0.4831	0.9831	−0.3262	0.024*	
C26	0.3367 (3)	0.9305 (2)	−0.26774 (19)	0.0168 (6)	
C27	0.2930 (3)	0.8929 (2)	−0.1943 (2)	0.0204 (7)	
H27	0.2129	0.8736	−0.1906	0.025*	
C28	0.3677 (3)	0.8836 (2)	−0.1253 (2)	0.0197 (7)	
H28	0.3383	0.8581	−0.0743	0.024*	

### Atomic displacement parameters (Å^2^)

**Table:**

	*U*^11^	*U*^22^	*U*^33^	*U*^12^	*U*^13^	*U*^23^
Br1	0.02349 (14)	0.02282 (14)	0.01741 (15)	−0.00056 (13)	−0.00417 (13)	0.00095 (13)
S1	0.0157 (3)	0.0244 (4)	0.0145 (4)	−0.0021 (3)	0.0021 (3)	0.0024 (3)
F1	0.0288 (10)	0.0224 (9)	0.0372 (12)	−0.0012 (9)	−0.0056 (10)	0.0159 (9)
N1	0.0152 (12)	0.0186 (11)	0.0117 (13)	−0.0003 (10)	−0.0012 (10)	−0.0026 (10)
N2	0.0187 (12)	0.0181 (11)	0.0153 (14)	0.0005 (10)	0.0004 (11)	−0.0009 (11)
N3	0.0176 (12)	0.0168 (11)	0.0119 (12)	0.0000 (11)	−0.0006 (11)	0.0005 (10)
N4	0.0181 (12)	0.0197 (12)	0.0137 (14)	0.0004 (11)	−0.0021 (11)	0.0045 (11)
N5	0.0126 (11)	0.0257 (13)	0.0128 (13)	−0.0008 (10)	0.0031 (10)	0.0055 (11)
N6	0.0126 (11)	0.0174 (12)	0.0166 (13)	0.0018 (10)	0.0022 (10)	0.0014 (10)
C1	0.0150 (13)	0.0222 (13)	0.0130 (14)	−0.0009 (13)	−0.0013 (13)	0.0050 (11)
C2	0.0214 (14)	0.0231 (13)	0.0178 (15)	0.0020 (12)	−0.0002 (16)	−0.0005 (13)
C3	0.0273 (17)	0.0338 (18)	0.0153 (17)	−0.0043 (15)	−0.0011 (14)	−0.0030 (14)
C4	0.0226 (16)	0.0355 (18)	0.0161 (17)	−0.0044 (15)	−0.0018 (13)	0.0095 (15)
C5	0.0236 (15)	0.0221 (15)	0.028 (2)	0.0002 (14)	−0.0025 (14)	0.0073 (14)
C6	0.0188 (14)	0.0219 (15)	0.0204 (17)	−0.0012 (13)	−0.0020 (13)	−0.0002 (13)
C7	0.033 (2)	0.057 (2)	0.020 (2)	−0.0065 (19)	−0.0102 (17)	0.0115 (18)
C8	0.0159 (13)	0.0236 (15)	0.0139 (15)	0.0004 (12)	0.0026 (12)	0.0021 (13)
C9	0.0143 (14)	0.0143 (14)	0.0166 (16)	−0.0011 (12)	0.0012 (12)	−0.0013 (12)
C10	0.0175 (13)	0.0129 (13)	0.0123 (15)	−0.0016 (12)	0.0007 (12)	0.0018 (11)
C11	0.0160 (13)	0.0145 (13)	0.0109 (15)	−0.0034 (12)	0.0020 (12)	−0.0032 (12)
C12	0.0147 (14)	0.0203 (15)	0.0161 (16)	0.0012 (12)	0.0024 (13)	0.0046 (13)
C13	0.0146 (13)	0.0171 (14)	0.0128 (15)	−0.0004 (12)	0.0007 (12)	0.0010 (12)
C14	0.0116 (13)	0.0197 (14)	0.0165 (17)	−0.0018 (12)	−0.0034 (12)	0.0045 (12)
C15	0.0190 (14)	0.0231 (15)	0.0167 (17)	0.0005 (13)	0.0012 (13)	0.0015 (13)
C16	0.0200 (14)	0.0212 (15)	0.031 (2)	0.0050 (12)	−0.0010 (16)	−0.0028 (16)
C17	0.0183 (15)	0.0195 (15)	0.0244 (18)	−0.0034 (13)	−0.0075 (14)	0.0130 (14)
C18	0.0135 (14)	0.0278 (16)	0.0152 (16)	−0.0032 (13)	−0.0026 (13)	0.0070 (14)
C19	0.0140 (14)	0.0199 (14)	0.0191 (17)	0.0012 (12)	−0.0035 (13)	−0.0010 (13)
C20	0.0152 (14)	0.0159 (12)	0.0146 (14)	0.0030 (12)	0.0020 (12)	0.0017 (11)
C21	0.0154 (14)	0.0218 (15)	0.0198 (17)	−0.0011 (13)	0.0004 (13)	0.0017 (14)
C22	0.0163 (14)	0.0161 (14)	0.0190 (17)	0.0017 (12)	−0.0014 (13)	−0.0002 (13)
C23	0.0152 (13)	0.0138 (12)	0.0175 (16)	0.0025 (11)	0.0003 (13)	0.0018 (13)
C24	0.0150 (13)	0.0164 (14)	0.0226 (16)	−0.0011 (13)	0.0009 (12)	0.0011 (14)
C25	0.0217 (14)	0.0193 (15)	0.0180 (16)	0.0031 (13)	0.0014 (13)	0.0037 (13)
C26	0.0184 (14)	0.0163 (14)	0.0158 (15)	0.0043 (13)	−0.0044 (12)	−0.0001 (12)
C27	0.0145 (15)	0.0253 (16)	0.0215 (17)	−0.0012 (12)	0.0009 (13)	−0.0001 (13)
C28	0.0185 (14)	0.0243 (15)	0.0161 (18)	0.0005 (12)	0.0029 (13)	0.0016 (13)

### Geometric parameters (Å, º)

**Table:**

Br1—C26	1.901 (3)	C8—H8C	0.9800
S1—C21	1.728 (3)	C9—C10	1.381 (4)
S1—C20	1.734 (3)	C10—C11	1.445 (4)
F1—C17	1.367 (3)	C11—C12	1.501 (4)
N1—C9	1.358 (4)	C12—C13	1.535 (4)
N1—N2	1.367 (3)	C12—H12A	0.9900
N1—C1	1.439 (4)	C12—H12B	0.9900
N2—N3	1.300 (3)	C13—C14	1.521 (4)
N3—C10	1.363 (4)	C13—H13	1.0000
N4—C11	1.290 (4)	C14—C19	1.389 (4)
N4—N5	1.397 (3)	C14—C15	1.385 (4)
N5—C20	1.381 (4)	C15—C16	1.402 (4)
N5—C13	1.475 (4)	C15—H15	0.9500
N6—C20	1.291 (4)	C16—C17	1.365 (5)
N6—C22	1.394 (4)	C16—H16	0.9500
C1—C6	1.384 (4)	C17—C18	1.376 (4)
C1—C2	1.390 (4)	C18—C19	1.380 (4)
C2—C3	1.377 (4)	C18—H18	0.9500
C2—H2	0.9500	C19—H19	0.9500
C3—C4	1.390 (5)	C21—C22	1.357 (4)
C3—H3	0.9500	C21—H21	0.9500
C4—C5	1.388 (5)	C22—C23	1.476 (4)
C4—C7	1.504 (4)	C23—C24	1.392 (4)
C5—C6	1.383 (4)	C23—C28	1.400 (4)
C5—H5	0.9500	C24—C25	1.383 (4)
C6—H6	0.9500	C24—H24	0.9500
C7—H7A	0.9800	C25—C26	1.390 (4)
C7—H7B	0.9800	C25—H25	0.9500
C7—H7C	0.9800	C26—C27	1.378 (4)
C8—C9	1.480 (4)	C27—C28	1.396 (4)
C8—H8A	0.9800	C27—H27	0.9500
C8—H8B	0.9800	C28—H28	0.9500
			
C21—S1—C20	87.81 (15)	C13—C12—H12B	111.3
C9—N1—N2	112.0 (2)	H12A—C12—H12B	109.2
C9—N1—C1	128.1 (3)	N5—C13—C14	113.2 (2)
N2—N1—C1	119.9 (2)	N5—C13—C12	101.5 (2)
N3—N2—N1	106.3 (2)	C14—C13—C12	113.6 (2)
N2—N3—C10	109.5 (2)	N5—C13—H13	109.4
C11—N4—N5	108.0 (2)	C14—C13—H13	109.4
C20—N5—N4	116.8 (2)	C12—C13—H13	109.4
C20—N5—C13	121.5 (3)	C19—C14—C15	119.2 (3)
N4—N5—C13	112.3 (2)	C19—C14—C13	117.9 (3)
C20—N6—C22	109.0 (2)	C15—C14—C13	122.9 (3)
C6—C1—C2	121.2 (3)	C14—C15—C16	120.5 (3)
C6—C1—N1	119.6 (3)	C14—C15—H15	119.7
C2—C1—N1	119.1 (3)	C16—C15—H15	119.7
C3—C2—C1	118.6 (3)	C17—C16—C15	118.1 (3)
C3—C2—H2	120.7	C17—C16—H16	120.9
C1—C2—H2	120.7	C15—C16—H16	120.9
C2—C3—C4	121.6 (3)	C16—C17—F1	119.0 (3)
C2—C3—H3	119.2	C16—C17—C18	122.8 (3)
C4—C3—H3	119.2	F1—C17—C18	118.2 (3)
C3—C4—C5	118.5 (3)	C17—C18—C19	118.4 (3)
C3—C4—C7	120.8 (3)	C17—C18—H18	120.8
C5—C4—C7	120.8 (3)	C19—C18—H18	120.8
C6—C5—C4	121.2 (3)	C14—C19—C18	120.9 (3)
C6—C5—H5	119.4	C14—C19—H19	119.6
C4—C5—H5	119.4	C18—C19—H19	119.6
C5—C6—C1	119.0 (3)	N6—C20—N5	122.9 (3)
C5—C6—H6	120.5	N6—C20—S1	117.1 (2)
C1—C6—H6	120.5	N5—C20—S1	120.0 (2)
C4—C7—H7A	109.5	C22—C21—S1	110.5 (2)
C4—C7—H7B	109.5	C22—C21—H21	124.7
H7A—C7—H7B	109.5	S1—C21—H21	124.7
C4—C7—H7C	109.5	C21—C22—N6	115.6 (3)
H7A—C7—H7C	109.5	C21—C22—C23	127.2 (3)
H7B—C7—H7C	109.5	N6—C22—C23	117.1 (3)
C9—C8—H8A	109.5	C24—C23—C28	118.9 (3)
C9—C8—H8B	109.5	C24—C23—C22	119.9 (3)
H8A—C8—H8B	109.5	C28—C23—C22	121.2 (3)
C9—C8—H8C	109.5	C25—C24—C23	121.2 (3)
H8A—C8—H8C	109.5	C25—C24—H24	119.4
H8B—C8—H8C	109.5	C23—C24—H24	119.4
N1—C9—C10	102.8 (3)	C24—C25—C26	118.9 (3)
N1—C9—C8	123.8 (3)	C24—C25—H25	120.6
C10—C9—C8	133.4 (3)	C26—C25—H25	120.6
N3—C10—C9	109.4 (3)	C27—C26—C25	121.4 (3)
N3—C10—C11	120.3 (3)	C27—C26—Br1	119.2 (2)
C9—C10—C11	130.2 (3)	C25—C26—Br1	119.4 (2)
N4—C11—C10	123.3 (3)	C26—C27—C28	119.2 (3)
N4—C11—C12	114.0 (3)	C26—C27—H27	120.4
C10—C11—C12	122.7 (3)	C28—C27—H27	120.4
C11—C12—C13	102.6 (2)	C27—C28—C23	120.4 (3)
C11—C12—H12A	111.3	C27—C28—H28	119.8
C13—C12—H12A	111.3	C23—C28—H28	119.8
C11—C12—H12B	111.3		
			
C9—N1—N2—N3	0.7 (3)	C11—C12—C13—C14	132.8 (3)
C1—N1—N2—N3	−177.6 (2)	N5—C13—C14—C19	−169.6 (3)
N1—N2—N3—C10	−0.4 (3)	C12—C13—C14—C19	75.3 (3)
C11—N4—N5—C20	156.5 (3)	N5—C13—C14—C15	13.2 (4)
C11—N4—N5—C13	9.4 (3)	C12—C13—C14—C15	−101.9 (3)
C9—N1—C1—C6	118.3 (3)	C19—C14—C15—C16	1.3 (4)
N2—N1—C1—C6	−63.7 (4)	C13—C14—C15—C16	178.5 (3)
C9—N1—C1—C2	−64.7 (4)	C14—C15—C16—C17	−0.5 (5)
N2—N1—C1—C2	113.3 (3)	C15—C16—C17—F1	179.1 (3)
C6—C1—C2—C3	0.7 (5)	C15—C16—C17—C18	−1.0 (5)
N1—C1—C2—C3	−176.2 (3)	C16—C17—C18—C19	1.7 (5)
C1—C2—C3—C4	0.9 (5)	F1—C17—C18—C19	−178.5 (3)
C2—C3—C4—C5	−2.0 (5)	C15—C14—C19—C18	−0.7 (4)
C2—C3—C4—C7	176.2 (3)	C13—C14—C19—C18	−178.0 (3)
C3—C4—C5—C6	1.6 (5)	C17—C18—C19—C14	−0.8 (4)
C7—C4—C5—C6	−176.6 (3)	C22—N6—C20—N5	−178.7 (3)
C4—C5—C6—C1	0.0 (5)	C22—N6—C20—S1	−0.4 (3)
C2—C1—C6—C5	−1.2 (5)	N4—N5—C20—N6	−166.6 (3)
N1—C1—C6—C5	175.8 (3)	C13—N5—C20—N6	−22.6 (4)
N2—N1—C9—C10	−0.7 (3)	N4—N5—C20—S1	15.2 (4)
C1—N1—C9—C10	177.5 (3)	C13—N5—C20—S1	159.2 (2)
N2—N1—C9—C8	178.3 (3)	C21—S1—C20—N6	−0.3 (2)
C1—N1—C9—C8	−3.5 (5)	C21—S1—C20—N5	178.0 (2)
N2—N3—C10—C9	−0.1 (3)	C20—S1—C21—C22	1.0 (2)
N2—N3—C10—C11	178.7 (2)	S1—C21—C22—N6	−1.5 (3)
N1—C9—C10—N3	0.5 (3)	S1—C21—C22—C23	176.9 (2)
C8—C9—C10—N3	−178.4 (3)	C20—N6—C22—C21	1.2 (4)
N1—C9—C10—C11	−178.2 (3)	C20—N6—C22—C23	−177.4 (2)
C8—C9—C10—C11	2.9 (6)	C21—C22—C23—C24	176.1 (3)
N5—N4—C11—C10	176.4 (3)	N6—C22—C23—C24	−5.6 (4)
N5—N4—C11—C12	−1.1 (3)	C21—C22—C23—C28	−6.0 (5)
N3—C10—C11—N4	178.4 (3)	N6—C22—C23—C28	172.4 (3)
C9—C10—C11—N4	−3.1 (5)	C28—C23—C24—C25	0.3 (4)
N3—C10—C11—C12	−4.3 (4)	C22—C23—C24—C25	178.3 (3)
C9—C10—C11—C12	174.2 (3)	C23—C24—C25—C26	0.3 (5)
N4—C11—C12—C13	−6.8 (3)	C24—C25—C26—C27	−0.8 (5)
C10—C11—C12—C13	175.6 (2)	C24—C25—C26—Br1	179.5 (2)
C20—N5—C13—C14	79.6 (3)	C25—C26—C27—C28	0.8 (4)
N4—N5—C13—C14	−135.1 (2)	Br1—C26—C27—C28	−179.5 (2)
C20—N5—C13—C12	−158.3 (3)	C26—C27—C28—C23	−0.3 (4)
N4—N5—C13—C12	−12.9 (3)	C24—C23—C28—C27	−0.3 (4)
C11—C12—C13—N5	11.0 (3)	C22—C23—C28—C27	−178.2 (3)

### Hydrogen-bond geometry (Å, º)

Cg1 is the centroid of the C23–C28 benzene ring.

**Table:**

*D*—H···*A*	*D*—H	H···*A*	*D*···*A*	*D*—H···*A*
C13—H13···N2^i^	1.00	2.58	3.488 (4)	151
C27—H27···F1^ii^	0.95	2.53	3.358 (4)	146
C8—H8*A*···*Cg*1^iii^	0.98	2.84	3.401 (3)	117

Symmetry codes: (i) *x*−1/2, −*y*+3/2, −*z*; (ii) −*x*+1, *y*−1/2, −*z*−1/2; (iii) −*x*, *y*+3/2, −*z*+1/2.
